# Therapeutic Exercise for Hospitalized Sarcopenic Patients: A Systematic Review and Meta-Analysis

**DOI:** 10.3390/sports13090326

**Published:** 2025-09-12

**Authors:** Olivier Chan-Fook, Javier Martin-Núñez, Julia Raya-Benítez, Alba Navas-Otero, Irene Cabrera-Martos, Marie Carmen Valenza, Alejandro Heredia-Ciuró

**Affiliations:** 1 Centre Europeen d’Enseignement en Reeducation et Readaptation Fonctionnelle, 93206 Saint-Denis, France; 2 Department of Physiotherapy, Faculty of Health Sciences, University of Granada, 18012 Granada, Spain; javimn@ugr.es (J.M.-N.); albanavas@ugr.es (A.N.-O.); irenecm@ugr.es (I.C.-M.); ahc@ugr.es (A.H.-C.); 3 Department of Nursing, Faculty of Health Sciences, University of Granada, 18012 Granada, Spain; juliarb@ugr.es

**Keywords:** sarcopenia, hospitalization, therapeutic exercise, muscle strength, physical performance, cognitive function

## Abstract

Sarcopenia is a progressive and generalized skeletal muscle disorder associated with an impairment of functional status, increasing dependency and mortality. The high prevalence among hospitalized patients has increased interest in active interventions such as exercise; however, the effectiveness of therapeutic exercise in this population remains unclear. This systematic review with a meta-analysis aims to evaluate the effectiveness of therapeutic exercises in hospitalized patients diagnosed with or at risk of sarcopenia. A systematic search was conducted in Medline, Web of Science, and Scopus databases following PRISMA guidelines. Randomized controlled trials assessing therapeutic exercises for sarcopenic or at-risk hospitalized patients were included. Methodological quality was evaluated using the TIDieR Checklist and the ROB2 tool. We performed a meta-analysis addressing muscle strength, physical performance and cognitive function. Six studies met the inclusion criteria, with a total of 1468 participants. Similar interventions were observed, including mainly resistance and balance exercises. Therapeutic exercises demonstrated significant improvements in physical performance (2.98 (1.13–4.83); *p* = 0.002; I^2^ = 99%), muscle strength (2.11 (0.20–4.01); *p* = 0.03; I^2^ = 99%) and cognitive function (0.77 (0.25–1.29); *p* = 0.004; I^2^ = 98%) across several studies. Therapeutic exercises appear to improve sarcopenic outcomes in hospitalized patients, supporting their role as a non-pharmacological intervention to mitigate sarcopenia-related complications. However, due to the lack of reported muscle mass outcomes, as well as the limited number and methodological quality of the included studies, further well-designed trials are needed to confirm these findings.

## 1. Introduction

Sarcopenia is emerging as a major public health concern due to its rapidly increasing incidence associated with ageing and chronic diseases [1]. By 2045, its prevalence is projected to reach up to 22.3%, which would correspond to more than 10 million cases in Europe alone, in parallel with demographic shifts [1]. In this context, research into sarcopenia is prioritized due to its health and socioeconomic burden [1].

Sarcopenia is characterized by a progressive and generalized decline in muscle mass and function, which is associated with an increased risk of falls, fractures, and loss of autonomy [2]. This ongoing deterioration in physical capacity contributes to increased frailty and morbidity [3,4], impairing activities of daily living and patients’ quality of life [5]. All of these factors link sarcopenia to an elevated risk of hospitalization [6] and mortality [7].

Hospitalization also represents a high risk factor for sarcopenia. Hospitalized patients often experience abrupt changes in environment, autonomy, dietary and physical activity habits, all of which accelerate the onset and progression of sarcopenia [8]. More than 10% of hospitalized patients develop this condition, being more likely to experience prolonged hospitalization, complications, and increased mortality [2]. Moreover, sarcopenia often form part of a dual decline with cognitive impairment during hospitalization [9], linked to greater functional deterioration and additional increased risk of complications and mortality [8,9,10].

Therefore, the published literature underscores the need to manage hospital-associated sarcopenia [11]. Current sarcopenia treatment include nutritional, pharmacological and physical approaches such as protein supplementation [12], myostatin inhibitors [13], and neuromuscular electrical stimulation [14]. Among them, therapeutic exercise remains the most effective and evidence-based intervention. It has been extensively studied in patients with sarcopenia, with consistent improvements reported in muscle mass, strength, and functional performance [15,16]. It also has proven benefits, enhancing balance, reducing fall risk, and significantly improving quality of life [16,17].

Although its benefits are currently well established, the application in hospitalized patients warrants specific attention, as tailoring interventions to individual needs is essential to ensure optimal outcomes following exercise [15,17,18]. The broad scientific literature highlights the particular vulnerabilities and clinical characteristics of this high-risk subgroup, which must be carefully considered when designing therapeutic programs [18,19].

For this reason, this review aimed to evaluate the effects of therapeutic exercise on muscle strength, muscle mass, physical performance, and cognitive function in hospitalized patients with or at risk of sarcopenia.

## 2. Materials and Methods

### 2.1. Study Registration

This systematic review was conducted in accordance with the Cochrane Handbook for Systematic Reviews of Interventions [20] and followed the guidelines outlined in the Preferred Reporting Items for Systematic Reviews and Meta-Analyses (PRISMA) statement [21] (Table S3). The review protocol was registered with the International Prospective Register of Systematic Reviews (PROSPERO) under the ID CRD42024607072 (Date: 8 November 2024).

### 2.2. Search Strategy

A systematic literature search was conducted across the following databases: MEDLINE (via PubMed), Web of Science, and Scopus, covering articles published from database inception to August 2025. The search strategy for MEDLINE was developed through (1) a comprehensive review of the MeSH database, (2) identification of relevant keywords based on prior systematic reviews, and (3) consultation with an information specialist.

The search strategy was rigorously tested and refined to maximize sensitivity and specificity, then adapted for use in each database (Table S1). Additionally, the reference lists of included articles and relevant reviews were manually screened to identify any potentially eligible studies not captured by the initial search.

To structure the research question, we used the PICOS framework [22] (Participants, Interventions, Comparisons, Outcomes, and Study design): P) Hospitalized patients with or at risk of sarcopenia; I) Therapeutic exercise; C) Any intervention without therapeutic exercise, or no intervention; O) Sarcopenia-related outcomes; and S) Randomized controlled trials (RCTs) and pilot RCTs.

The heterogeneous clinical presentation of sarcopenia has historically complicated its characterization. Therefore, this review aligned with the most recent criteria proposed by the European Working Group on Sarcopenia in Older People, version 2 (EWGSOP2, 2018) [23,24], which includes a structured diagnostic algorithm and definitions of sarcopenia-related outcomes.

The first step is to identify which patients are likely to develop or potentially already have sarcopenia [23,24]. The initial suspicion and diagnosis of sarcopenia are guided by measurements of muscle strength, alongside qualitative and quantitative assessments of muscle mass [23,24]. Severity is then categorized based on physical performance tests, which also provide a global assessment of functional status and autonomy [23,24]. Patients falling below the EWGSOP2-defined thresholds in at least one criterion were considered at risk of sarcopenia. A range of validated tools was accepted for these assessments, depending on the clinical context of each study.

### 2.3. Exclusion Criteria

Studies were excluded if they involved cancer patients or patients with severe neurological disorders or cognitive impairments that prevented them from understanding basic instructions, or if the patients resided in long-term care facilities (e.g., nursing homes or residential institutions). We also excluded studies for which the full text was not available or those published in languages other than English, Spanish, or French based on our team’s language proficiency for ensuring accurate data extraction and interpretation.

### 2.4. Study Selection

After retrieving the databases records, duplicates were removed, and titles, abstracts, and full texts were screened for eligibility. To minimize selection bias, two investigators and independently screened the articles. Disagreements were resolved through discussion with a third investigator. Following study selection, data extraction and quality assessment were performed.

### 2.5. Data Extraction

Data extraction was carried out independently by two investigators using a standardized data collection form based on the Cochrane Handbook for Systematic Reviews [20]. Extracted data included: authors, publication year, country, study design, sample size, sex distribution, mean age, intervention details, study duration, and reported outcomes. When available, clinical information such as the reason for hospitalization was also extracted. All data were investigator by a third author, and discrepancies were resolved through consensus.

### 2.6. Quality Assessment

The risk of bias in the included studies was assessed using the Cochrane Risk-of-Bias tool version 2.0 (RoB-2) [25], evaluating five domains: the randomization process, deviations from intended interventions, missing outcome data, outcome measurement, and selection of the reported results. Studies were rated as having low, some concerns, or high risk of bias. Assessments were conducted using the official RoB-2 Excel template.

Additionally, the quality of reporting for exercise interventions was evaluated using the Template for Intervention Description and Replication (TIDieR) checklist [26]. This 12-item tool enhances reproducibility by assessing the completeness of intervention descriptions. Each item was scored on a 3-point Likert scale, 0 (not reported), 1 (partially reported), and 2 (adequately reported), for a total score ranging from 0 to 24 [27]. Based on prior literature, interventions descriptions were classified as good (≥21), moderate (18–20), or poor (≤17) [28].

### 2.7. Meta-Analysis

Quantitative synthesis was performed using Review Manager (RevMan version 5.4, 2020) for all pilot and full RCTs reporting post-intervention means and standard deviations for muscle strength, physical performance, or cognitive function. Mean values, standard deviations, and sample sizes were extracted for each treatment arm to calculate overall mean differences.

When multiple follow-up points were reported, only the data collected at the end of the intervention were used. If necessary data (e.g., means or standard deviations) were missing, authors were contacted. If standard deviations were not reported but *p*-values or 95% confidence intervals were available, these were used to calculate missing values using RevMan’s built-in calculator.

For continuous outcomes, weighted mean differences (MDs) were used when all studies applied the same measurement scale, and standardized mean differences (SMDs) were used when all scales were assumed to measure the same underlying condition, but some studies measured the outcomes on different scales. All results are reported with 95% confidence intervals.

Effect sizes were pooled using either fixed-effect or random-effects models depending on heterogeneity, assessed via the I^2^ statistic. Fixed-effect models were applied when I^2^ was below 50% [20]. Outlier detection was conducted through visual inspection of forest plots, and sensitivity analyses were performed by removing studies with the most influence on the pooled effect size.

## 3. Results

### 3.1. Study Selection

**Figure 1:**
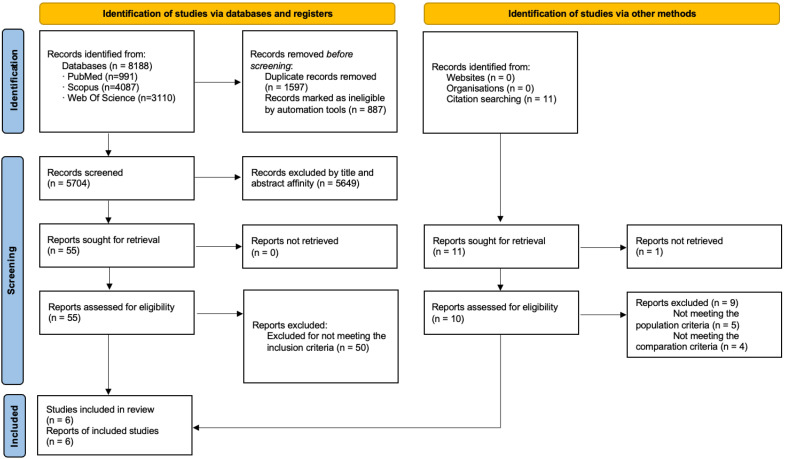
Flow Diagram.

A total of 8188 records were initially identified through database searches. After removing duplicates, 5704 titles remained for screening. Based on title and abstract screening, 55 full-text articles were assessed for eligibility. Of these, 50 were excluded for not meeting the inclusion criteria, resulting in 5 studies deemed eligible for inclusion. Eleven studies did not meet the population criteria, seventeen did not report the relevant outcome variables, and five did not compare two exercise groups, failing to meet the comparator criterion. Additionally, thirteen studies were not RCTs or pilot RCTs, and four studies included participants from long-term care facilities. One additional study was identified through manual reference searching. In total, 6 studies were included in both the qualitative and quantitative syntheses [29,30,31,32,33,34]. The PRISMA flow diagram is presented in Figure 1.

### 3.2. Study Characteristic

Table 1 summarizes the characteristics of the included studies and their corresponding risk of bias assessments. These data were independently extracted by two investigators. Two of the six studies were conducted in North America [30,34], while the remaining four were carried out in Europe [29,31,32,33]. Participants were recruited from various hospital units, including geriatric [29,31,32], cardiovascular [30], pulmonary [33], and intensive care units [34]. Most studies [29,30,31,32,33] included older adults aged 65 years or older; although the study by Morris et al. [34] included participants of any age.

Across the six studies, a total of 1468 patients were included, with roughly half allocated to the intervention group and half to the control group. Females accounted for 53.16% of the total sample (n = 780), and mean ages ranged from 55 to 87 years. The reasons for hospitalization were varied, with cardiovascular and pulmonary diseases being the most prevalent etiologies. Studies applied similar exclusion criteria, highlighting short hospital stays, cognitive impairment, inability to cooperate, terminal illness, uncontrolled heart disease, recent surgery, and bone fractures. 

**Figure 2:**
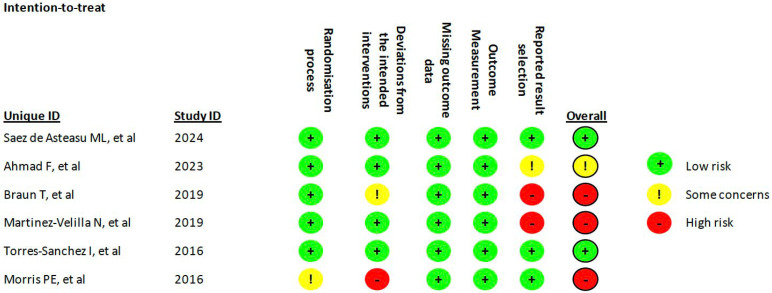
Risk of Bias assessment [29,30,31,32,33,34].

When assessed using the Cochrane Risk of Bias tool, three studies were classified as having a high risk of bias [31,32,34], and one study raised some concerns [30]. The studies by Sáez de Asteasu et al. [29] and Torres-Sánchez et al. [33] demonstrated a low risk of bias. The domains most frequently contributing to increased bias were “deviations from intended interventions” and “selection of the reported results.” Detailed risk of bias assessments are shown in Figure 2.

Table 2 presents the intervention details and study results. The quality of intervention reporting, as assessed using the TIDieR checklist, yielded total scores ranging from 18 to 22, indicating moderate to good reporting quality (Table S2).

Across the included studies [29,30,31,32,34], most interventions combined multiple exercise modalities. Moderate-intensity resistance training was reported in four studies [29,31,32,34], typically consisting of 2–3 sets of 8–15 repetitions, with intensity monitored through the Borg scale or percentage of one-repetition maximum. Balance-oriented activities, such as unipodal, bipodal, or walking exercises, were incorporated in five studies [29,30,31,32,34]. In addition, two studies [29,32] introduced unsupervised functional training, where participants performed joint mobility exercises with added external load. Torres-Sánchez et al. [33] applied cycle ergometer-based aerobic training at moderate intensity, whereas Ahmad et al. [30] described strength and flexibility exercises but did not provide specific dosage details.

Overall, session characteristics were relatively homogeneous: 20–30 min per session, performed 1–3 times daily, on 5–7 days per week, with program durations spanning from 1 to 3 weeks.

Control interventions consisted of standard medical care in all six studies [29,30,31,32,33,34], with conventional physiotherapy used in five [29,30,31,32,34]. Ahmad et al. [30] additionally provided nutritional and pharmacological management in the control group.

Significant improvements in muscle strength [29,33], physical performance [29,30,32], and cognitive function [29,32] were reported in favor of therapeutic exercise compared to usual care. However, Braun et al. [31] and Morris et al. [34] did not report statistically significant effects. Notably, none of the studies included outcomes related to muscle mass.

Concerning intervention safety, no adverse events were reported in most studies [29,30,31,32,33]. Only Morris et al. [34] noted a single episode of asymptomatic bradycardia during resistance training.

### 3.3. Results Obtained in Meta-Analysis

**Figure 3:**
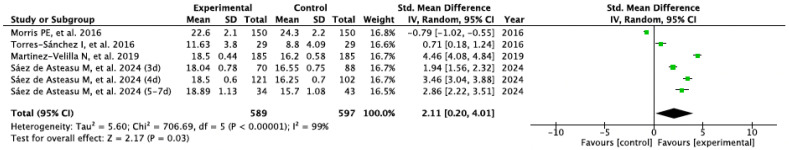
Muscle strength forest plot [29,32,33,34].

Figure 3 presents the meta-analysis for muscle strength outcomes. The pooled SMD demonstrated a significant overall effect of therapeutic exercise compared to control interventions (SMD = 2.11; 95% CI: 0.20–4.01; *p* = 0.03). Heterogeneity was high (I^2^ = 99%) and not attributable to chance.

**Figure 4:**
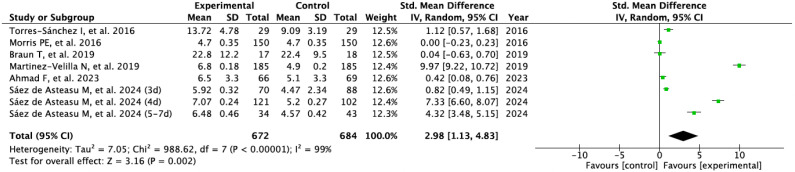
Physical performance forest plot [29,30,31,32,33,34].

Results for physical performance are shown in Figure 4. The pooled SMD indicated a significant benefit of therapeutic exercise over control (SMD = 2.98; 95% CI: 1.13–4.83; *p* = 0.002). Heterogeneity was also high (I^2^ = 99%), not attributable to chance.

**Figure 5:**
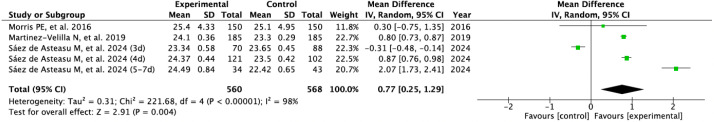
Cognitive function forest plot [29,32,34].

Cognitive outcomes were analyzed using Mini-Mental State Examination (MMSE) scores. As shown in Figure 5, the pooled MD favored therapeutic exercise over control (MD = 0.77; 95% CI: 0.25–1.29; *p* = 0.004). Again, heterogeneity was high (I^2^ = 98%) and not attributable to chance.

Concerning to sensitivity analyses, the results indicate that the robustness of our conclusions was not affected by the sensitivity analyses. Specifically, the associations remained statistically significant for muscle strength (*p* = 0.004), physical performance (*p* < 0.0001), and cognitive function (*p* = 0.003).

## 4. Discussion

This study aimed to explore the effectiveness of therapeutic exercise in hospitalized patients diagnosed with or at risk of sarcopenia. Our findings indicate beneficial effects of applying therapeutic exercise in these patients, with significant improvements in muscle strength and physical performance as main sarcopenia-related outcomes. Additionally, cognitive function also showed significant benefits compared to usual care. These results are in line with previous studies in sarcopenic patients, reporting similar benefits to ours when applied resistance training [19]. In this regards, integrating therapeutic exercise into routine hospital care could help reduce the musculoskeletal and cognitive deterioration during hospitalization, which could reduce associated healthcare and social impact.

These findings suggest that exercise interventions may have the potential to reduce healthcare burden; however, this remains a hypothesis that requires confirmation through dedicated economic evaluations.

The sample characteristics in this review were similar to those in previous systematic reviews of hospitalized sarcopenic patients [6]. These individuals typically present with three key vulnerability factors [35]: (1) sociodemographic aspects (including advanced age, high body mass index, or limitations in activities of daily living); (2) comorbidities (notably cardiovascular and respiratory conditions); and (3) behavioral factors (sedentary lifestyle, poor nutrition, and inadequate sleep…), with hospitalization playing a significant role in exacerbating these factors and increasing the risk of sarcopenia [36].

Concerning the interventions, most of the studies presented a similar structure, with resistance and balance exercise as the main components. This approach aligns with the principles of the VIVIFRAIL program, which has been widely implemented in frail older populations [37]. Its core components (progressive resistance, balance, and mobility training) together with the structured exercise dose, intensity, and progression tailored to individual functional capacity, closely mirror the principles of the interventions applied in the included studies. This exercise combination has been strongly advocated since it appears to improve muscular performance, making it possible to stimulate gains in muscle strength, joint mobility, balance, performance and quality of life [16,17].

There was also high consistency in exercise dosage across studies. Most protocols involved short sessions repeated throughout the day, performed at least five times per week and at moderate intensity. Similar parameters have been used effectively in other hospitalized populations, producing comparable improvements in strength and physical performance [38]. This frequency and intensity of exercise have proved to optimize functional gains while minimize the risk of falls, an essential objective in managing sarcopenia [14,15].

These findings are further supported by the outcomes observed following the implementation of the interventions. They were associated with a low incidence of adverse effects. It was to be expected since exercise therapy, when properly dosed and supervised, has been shown to be an effective and safe intervention across a wide range of clinical populations [39,40].

The meta-analyzed results in sarcopenia-related outcomes are in line with the results of similar reviews in sarcopenic patients, where the application of therapeutic exercise also reported beneficial effects in muscle strength, physical performance and muscle mass [41]. These benefits help counteract the complex interplay of contributing factors, such as systemic inflammation [42], immobilization, and undernutrition, which disrupt muscle protein synthesis and accelerate muscle wasting and sarcopenia progression [5,43]. However, the absence of muscle mass data may limit the ability to fully understand the impact of interventions on sarcopenia, emphasizing the importance of standardized reporting in future research.

The findings related to cognitive function represent a particular result. Prior research has established a bidirectional relationship between sarcopenia and cognitive decline, including dementia. It has been associated with the decreased secretion of neurotrophic factors in sarcopenic states, which negatively affects brain function [9].

Exercise appears to improve cognitive performance through the muscle–brain axis, whereby skeletal muscle releases brain-derived neurotrophic factors that promote neuroplasticity, neurogenesis, and overall brain health [44]. Recently, Gao et al. [45] also reported that exercise-driven secretion of myokines such us BDNF, irisin, CTSB, IL-6, and IGF-1, enhances cognitive and muscular function. This has been further reinforced by Wang et al. [46], who emphasized that exercise type, intensity, and duration influence myokine expression and their cross-talk effects. Within this framework, our findings are particularly relevant, as the coexistence of physical and cognitive decline has been shown to predict higher morbidity and mortality rates in hospitalized patients [9].

The high heterogeneity observed in our meta-analysis was a concern for us. This variability likely reflects differences in intervention protocols and in the methods used to collect outcome measures, particularly in the studies by Morris PE et al. [34] and Martinez-Velilla N et al. [32]. Importantly, sensitivity analyses confirmed the robustness of our results, providing confidence in the reliability of the findings and strengthening the validity of our conclusions.

### Limitations

Some limitations should be acknowledged. This analysis includes a relatively small number of studies, which limited the possibility of conducting subgroup analyses and may have affected the robustness of the results. Subgroup analyses by age groups could have provided further insights into the potential influence of participant age on the outcomes. Broader inclusion criteria might have allowed the inclusion of additional studies. Limiting the review to studies published in English, Spanish, or French may have also introduced a potential language bias.

In addition, several of the included studies presented methodological weaknesses and a high risk of bias, warranting caution when interpreting the results. These factors highlight the need for larger, well-designed trials to strengthen the evidence base in this area. Nevertheless, previous reviews on sarcopenic populations have been conducted with a similar number of trials [4] and reported relevant findings.

Finally, none of the studies included in this systematic review reported muscle mass outcomes, despite muscle mass is one of the three diagnostic criteria for sarcopenia, so it could potentially affect the generalizability of our conclusions concerning to the application of the intervention in sarcopenic outcomes. However, current clinical perspectives increasingly prioritize muscle function over muscle mass assessment, as it more directly correlates with quality of life. Physical performance tests better reflect patients’ ability to perform activities of daily living, and muscle strength has been shown to be a strong prognostic factor for both fall risk and mortality [47,48]. A major advantage of these functional assessment criteria is their feasibility, being easily standardized, reproducible, cost-effective, and widely applicable in clinical settings.

## 5. Conclusions

This systematic review and meta-analysis support the potential role of therapeutic exercise as a non-pharmacological intervention to mitigate sarcopenia-related complications in hospitalized patients diagnosed with or at risk of sarcopenia, demonstrating improvements in muscle strength and physical performance. Notably, the findings also highlight potential benefits for cognitive function, which is particularly relevant given the high risk of cognitive decline in this population. Nevertheless, due to the substantial heterogeneity and risk of bias among the included studies, these results should be interpreted with caution, and further high-quality research is needed to standardize exercise protocols and expand the findings of this review, providing a stronger foundation for clinical practice.

## Figures and Tables

**Table 1:** Characteristics of study populations.

Study ID	Setting	Population	N Age (% Females)	Reason of Hospitalization	Exclusion Criteria	Risk of Bias
Saéz de Asteasu ML et al., 2024 [29]	Hospital Geriatric Unit (Spain)	Elderly patients (>75 y)	570 EG: 87.4 ± 4.6 (50%) CG: 87.3 ± 5.1 (54%)	Cardiovascular (33%) Infectious (25%) Pulmonary (12%) Digestive (9%) Neurological (5%) Other (16%)	- Planned hospital stay < 6 days - Severe cognitive impairment - Inability to cooperate - Terminal illness - Uncontrolled heart disease - Major surgery < 3 months - Bone Fracture < 3 months	LOW
Ahmad F et al., 2023 [30]	Hospital Cardiovascular Unit (Canada)	Elderly Cardiovascular patients (>65 y)	135 EG: 78.2 ± 8.0 (53%) CG: 80.2 ± 7.3 (55%)	Cardiovascular (80%) Other (20%)	- Planned hospital stay < 3 days - Cardiac surgery < 3 days - Clinically or mental unstable - Neurological disease - Palliative care	SOME CONCERNS
Braun T et al., 2019 [31]	Hospital Geriatric Unit (Germany)	Elderly patients (>65 y)	35 EG: 78.6 ± 7.5 (76%) CG: 83.1 ± 7.4 (72%)	Musculoskeletal (34%) Cardiovascular (17%) Pulmonary (8.5%) Digestive (2.8%) Other (37.7%)	- Cognitive impairment - Inability to cooperate - Language barrier - Psychiatric condition - Palliative care - Medical restriction for exercise	HIGH
Martinez-Velilla N et al., 2019 [32]	Hospital Geriatric Unit (Spain)	Elderly patients (>75 y)	370 EG: 87.6 ± 4.6 (54%) CG: 87.1 ± 7.2 (59%)	Cardiovascular (35%) Infectious (18%) Pulmonary (13%) Digestive (10%) Neurological (5%) Other (19%)	- Planned hospital stay < 6 days - Severe cognitive impairment - Palliative care - Uncontrolled heart disease - Recent surgery - Bone fracture	HIGH
Torres-Sánchez I et al., 2016 [33]	Hospital Pulmonary Unit (Spain)	Frail Elderly patients (>65 y) with COPD	58 EG: 75.65 ± 6.25 (24%) CG: 72.12 ± 8.2 (31%)	Pulmonary (100%)	- Inability to cooperate - Cognitive impairment - Psychiatric condition - Neurological disorders - Musculoskeletal disorders - Organ failure - Cancer - Recent exacerbation of COPD	LOW
Morris PE et al., 2016 [34]	Medical Center Intensive Care Unit (USA)	Adult patients with acute respiratory failure	300 EG: 55 ± 17 (56%) CG: 58 ± 14 (54%)	Pulmonary (100%)	- Cognitive impairment - Obesity - Neurological disease - Bone fracture	HIGH

CG: Control Group; COPD: Chronic Obstructive Pulmonary Disease; EG: Experimental Group; y: years.

**Table 2:** Characteristics of interventions.

Study ID	Experimental Intervention	TIDIER	Control Intervention	Variable	Relevant Results
Saéz de Asteasu ML et al., 2024 [29]	· Usual Care · Therapeutic Exercise:	20	Usual Care · Medical care · can included PT	- Physical Performance (SPPB) - Muscle Strength (handgrip) - Gate Speed (GVT) - Cognitive Function (MMSE)	EG improved compared to CG (*p* < 0.001). The 4-day program showed most significant benefits in physical performance. The 4-day program showed most significant benefits in muscle strength and cognition. No adverse events
	Resistance Training From 2 × 10 sets 30% RM Day 1 to 3 × 8 sets 60% RM Day 5–7 (>10% RM/day) 2–3 sets × 8–10 reps Rises chair—Chess and leg press—Leg extension Balance Training Semi-tandem foot standing—Line walking—Stepping practice—Walking with obstacles—Unstable surfaces—Base of support variations—Leg Weight transfers Unsupervised Functional Training 0.5–1 kg anklets and handgrip ball Knee extension/flexion—Hip abduction—Walking 20 min session 2 sessions/day 3 to 7 days				
Ahmad F et al., 2023 [30]	· Usual Care · Cognitive stimulation · Protein supplementation · Anemia treatment · Therapeutic Exercise:	18	Usual Care · Medical care · PT 2–3 sessons/week · Nutritional care · Anemia treatment	- Physical Performance (SPPB) - Sarcopenia Screening (SARC-F)	EG improved physical performance (*p* < 0.001) and SARC-F (*p* < 0.02) compared to CG. No adverse events.
	Strength Training NR Flexibility Training NR Balance Training NR 20 min session 2 sessions/day				
Braun T et al., 2019 [31]	· Usual Care · Therapeutic Exercise:	22	Usual Care · Medical care · Multidisciplinary rehabilitation	- Physical Performance (TUG; 6 MWT) - Mobility (DEMMI & HABAM) - Walking Ability (Gate speed)	No statistically significant differences. No severe adverse events.
	Resistance Training 12–14/20 BORG 3 sets × 13–15 reps Progression individually directed by physiotherapist Chair rise—Heel raises—Partial squats—Stepping forward—Sideways up onto block Balance Training Clossed feet stance- Semitandem stance—Walking—Walking back—Stairclimbing 20–30 min session 4–5 sessions/week 1–3 weeks				
Martinez-Velilla N et al., 2019 [32]	· Usual Care · Therapeutic Exercise:	20	Usual Care · Medical care · PT (Walking exercises)	- Physical Performance (SPPB; Barthel) - Muscle Strength (handgrip) - Cognitive Function (MMSE)	CG showed impairment in physical performance after hospitalization, whereas EG reversed this trend (*p* < 0.001). Significant intervention benefits were also found at the cognitive function over the CG (*p* < 0.001). No adverse events.
	Resistance Training 30–60%RM 2–3 sets × 8–10 reps Progression Not Specified Squats rises chair—Leg press—Bilateral knee extension—Bench press Balance Training Semi-tandem foot standing—Line walking—Stepping practice—Walking with obstacles—Unstable surfaces—Base of support variations—Leg Weight transfers Unsupervised Functional Training 0.5–1 kg anklets and handgrip ball Knee extension/flexion—Hip abduction—Walking 20 min session 2 sessions/day 5–7 days				
Torres-Sánchez I et al., 2016 [33]	· Usual Care · Therapeutic Exercise:	18	Usual care · Medical care	- Physical Performance (30 STS) - Muscle Strength (quadriceps dynamometer) - Balance (One leg stance)	Significant between-group differences were observed in muscle strength (*p* = 0.028) and balance (*p* = 0.013) after the intervention. All the variables improved significantly (*p* < 0.05) in the EG. All the variables showed impairment in the CG. No adverse events.
	Aerobic Training + Oxygen Therapy Cycling with pedal increasing daily cycling intensity and progression adapted to the patients’ levels of dyspnea and fatigue (<6/10 BORG). 1 session/day 7 days/week During hospitalization				
Morris PE et al., 2016 [34]	· Usual Care · Conventional PT · Therapeutic Exercise:	18	Usual Care · Medical care · Weekly PT	- Physical Performance (SPPB; FPI) - Muscle Strength (handgrip; dynamometry) - Cognitive Function (MMSE)	No significant between group effects, but EG improve at follow-up with significant with-in group differences. No between differences in adverse events. There was an episode of asymptomatic bradycardia during resistance training.
	Resistance Training NR 3 sets × 8 reps Intensity maintained throughout intervention Joint mobility with elastic resistance bands, mainly focus on lower limbs Balance Training Seated balance—Forward and lateral weight shifting—Marching in place—Ambulation 3 sessions/day 7 days/week				

30 STS: 30 s sit to stand; 6 MWT: 6 min walking test; CG: Control Group; DEMMI: de Morton Mobility Index; EG: Experimental Group; SPPB: Short battery physical performance; TUG: Time up and go; NR: Not reported.

## Data Availability

Not applicable.

## Supplementary Materials

The following supporting information can be downloaded at: https://www.mdpi.com/article/10.3390/sports13090326/s1, Table S1: Search strategy; Table S2: TIDierR checklist; Table S3: PRISMA Checklist.

## Abbreviations

The following abbreviations are used in this manuscript:

RCT	Randomized Controlled Trial
PRISMA	Preferred Reporting Items for Systematic Reviews and Meta-Analyses
PROSPERO	Prospective Register of Systematic Reviews
EWGSOP	European Working Group on Sarcopenia in Older People
RoB-2	Cochrane Risk-of-Bias tool version 2.0
TIDieR	Template for Intervention Description and Replication
MD	Mean differences
SMD	Standardized mean differences
MMSE	Mini-Mental State Examination
